# Nurse practitioner/physician collaborative models of care: a scoping review protocol

**DOI:** 10.1186/s12877-023-03798-1

**Published:** 2023-02-16

**Authors:** Katherine S. McGilton, Lynn Haslam-Larmer, Aria Wills, Alexandra Krassikova, Jessica Babineau, Ben Robert, Carrie Heer, Carrie McAiney, Gail Dobell, Jennifer Bethell, Kelly Kay, Margaret Keatings, Sharon Kaasalainen, Sid Feldman, Souraya Sidani, Ruth Martin-Misener

**Affiliations:** 1 KITE Research Institute, Toronto Rehabilitation Institute – University Health Network, 550 University Avenue, Toronto, ON M5G 2A2 Canada; 2 Lawrence S. Bloomberg, Faculty of Nursing, University of Toronto, 155 College Street, Toronto, ON M5T 1P8 Canada; 3 Library & Information Services, University Health Network, 550 University Avenue, Toronto, ON M5G 2A2 Canada; 4 The Institute for Education Research, University Health Network, Toronto, Canada; 5 Perley Health, 1750 Russell Road, Ottawa, ON K1G 5Z6 Canada; 6 Faculty of Medicine, University of Ottawa, 451 Smyth Road #2044, Ottawa, ON K1H 8M5 Canada; 7 Brant Community Healthcare System, 200 Terrace Hill Street, Brantford, ON N3R 1G9 Canada; 8 Bruyère Research Institute, 85 Primrose Ave, Ottawa, ON K1R 6M1 Canada; 9 Schlegel-UW Research Institute for Aging, Waterloo, ON N2J 0E2 Canada; 10 School of Public Health Sciences, University of Waterloo, 200 University Avenue West, Waterloo, ON N2L 3G1 Canada; 11 Ontario Health, 130 Bloor Street West, Toronto, ON M5S 1N5 Canada; 12 Institute of Health Policy, Management and Evaluation, University of Toronto, 155 College Street, Toronto, ON M5T 3M7 Canada; 13 Provincial Geriatrics Leadership Ontario, Toronto, Canada; 14 Ontario Institute for Studies in Education and the Institute for Life Course and Aging, University of Toronto, 246 Bloor Street West, Toronto, ON M5S 1V4 Canada; 15 The Hospital for Sick Children, 555 University Ave, Toronto, ON M5G 1X8 Canada; 16 School of Nursing, McMaster University, 1280 Main Street West, Hamilton, ON L8S 4K1 Canada; 17 Baycrest Health Sciences, 3560 Bathurst Street, Toronto, ON M6A2E1 Canada; 18 Department of Family and Community Medicine, Temerty Medicine, University of Toronto, 1 King’s College Circle, Toronto, ON M5S 1A8 Canada; 19 Daphne Cockwell School of Nursing, Toronto Metropolitan University, 250 Victoria Street, Toronto, ON M5B 2K9 Canada; 20 School of Nursing, Dalhousie University, Room G26, Forrest Bldg., 5869 University Avenue, Halifax, NS B3H 4R2 Canada

**Keywords:** Nurse practitioner, Physician, Long-term care, Collaboration, Models of care, Knowledge synthesis, Scoping review

## Abstract

**Background:**

Before the COVID-19 pandemic, many long-term care (LTC) homes experienced difficulties in providing residents with access to primary care, typically delivered by community-based family physicians or nurse practitioners (NPs). During the pandemic, legislative changes in Ontario, Canada enabled NPs to act in the role of Medical Directors thereby empowering NPs to work to their full scope of practice. Emerging from this new context, it remains unclear how NPs and physicians will best work together as primary care providers. NP/physician collaborative models appear key to achieving optimal resident outcomes. This scoping review aims to map available evidence on existing collaborative models of care between NPs and physicians within LTC homes.

**Methods:**

The review will be guided by the research question, “What are the structures, processes and outcomes of collaborative models of care involving NPs and Physicians in LTC homes?” This scoping review will be conducted according to the methods framework for scoping reviews outlined by Arksey and O’Malley and refined by Levac et al., Colquhoun et al., and Daudt et al., as well as the Preferred Reporting Items for Systematic reviews and Meta-Analyses extension for Scoping Reviews (PRISMA-ScR) Statement. Electronic databases (MEDLINE, Embase + Embase Classic, APA PsycInfo, Cochrane Central Register of Controlled Trials, AMED, CINAHL, Ageline, and Scopus), grey literature, and reference lists of included articles will be searched. English language studies that describe NP and physician collaborative models within the LTC setting will be included.

**Discussion:**

This scoping review will consolidate what is known about existing NP/physician collaborative models of care in LTC homes. Results will be used to inform the development of a collaborative practice framework for long-term care clinical leadership.

**Supplementary Information:**

The online version contains supplementary material available at 10.1186/s12877-023-03798-1.

## Introduction

As the older adult population grows, worldwide the number of older adults who will require care in long-term care (LTC) homes will also increase. LTC homes provide 24/7 health and personal care support to residents with multiple health and social complexities including neurological, circulation, and musculoskeletal diseases, dementia, acute medical conditions, and stroke [1]. The pandemic impacted the LTC sector significantly, including the disproportionately high rate of COVID-19 deaths amongst LTC residents, the effects of social isolation on residents’ wellbeing, challenges with infection prevention and control practices, and staffing shortages due to limiting staff to single workplaces [2, 3]. This unprecedented situation illuminated long-standing deficiencies in LTC such as timely access to primary care for this complex, mainly older adult population [4]. The current model does not meet the needs of residents experiencing acute and chronic health issues, as limited accessibility to primary care providers is internationally cited as a common challenge [5–8].

There has been a steady increase in the number of NPs employed within the LTC sector over the last 20 years [9]. NPs are advanced practice nurses requiring graduate education for entry; their scope of practice include diagnosis, prescribing medications, and performing medical procedures [10]. Quality of care provided by NPs in LTC homes is comparable to that of physicians and has been shown to improve resident outcomes and reduce hospital transfers [11–13]. Additionally, NPs bring a breadth of advanced nursing skills to resident care, resulting in improved communication with family and care-partners, staff capacity-building, and strong clinical leadership and care coordination [11, 14].

The COVID-19 pandemic made in-person access to physicians increasingly difficult, as some physicians were recommended to adopt a “virtual-first” approach [15]. This experience provided an opportunity to examine the effects of optimizing the NP role and empowered NPs to work to their full scope of practice in a leadership role in LTC homes [16]. Several studies have reported positive outcomes from NP care during the pandemic, including successful COVID-19 recoveries, effective pain and symptom management, and ensuring dignified deaths for residents, demonstrating how NPs can provide urgently needed support to LTC residents [17, 18].

Similar to their physician colleagues, consultation and collaboration with other physicians and specialists is an essential component of the NP provider role; NPs do not function in isolation [3, 19, 20]. The intrinsic collaboration between NP and physician roles has resulted in a higher quality of resident care, decreased hospitalization rates, and the creation of an improved working environment for direct care providers [20]. Studies have investigated the abstract qualities inherent to the collaboration process. For optimal resident care, the NP-physician collaborative relationship requires commitment and time for development, where each care provider is able to employ qualities unique to their role [20]. When the collaborative process remains informal, it can result in an absence of funding to support collaborative practice models, inadequate educational preparation, and a lack of clarity regarding role definitions and the facilitators and barriers in implementing this process. The resources and capacities required to ensure successful collaboration have not yet been clearly elucidated. With some understanding of the underlying principles required for success, several collaborative models for the LTC context have been developed, such as the Evercare model and the Missouri Quality Initiative [21]. However, a comprehensive review and comparison of existing models has not been reported. Further, frameworks evaluating these models to inform best practices do not exist. While it is clear that residents, their care partners, and LTC care providers have benefitted from collaborative practices, a review of the existing models of care involving NP and physician collaboration is required to identify current gaps in the literature. A description of the structures, processes, and outcomes of models of NP-physician collaborative care will inform the development of a collaborative practice framework for LTC clinical leadership.

### Objective

This scoping review will examine and map evidence for collaborative models of care between NPs and physicians in LTC homes.

### Methodology

This scoping review will be conducted according to the methods framework for scoping reviews outlined by Arksey and O’Malley [22] and refined by Levac et al. [23], Colquhoun et al. [24], and Daudt et al. [25]. This framework is appropriate as the nature of the research question is exploratory, with the intention of mapping key concepts, evidence gaps, and implications. This scoping review will also be conducted according to the Preferred Reporting Items for Systematic reviews and Meta-Analyses extension for Scoping Reviews (PRISMA-ScR) Statement [26].

### Stage 1: Identifying the research questions

#### Primary question

- What are the structures, processes, and outcomes of collaborative models of care involving NPs and Physicians in LTC homes?

#### Secondary questions

- What are the strengths and weaknesses of each model?
- What are the barriers and facilitators for implementation of each model?
- How does each model impact resident, staff, health systems, and cost outcomes?

### Stage 2: Identifying relevant literature

We will conduct a comprehensive search of the scientific and grey literature as per scoping review methods [22–25]. We will implement a search strategy developed by our health sciences information specialist in conjunction with team members and key stakeholders. Prior to funding, a preliminary MEDLINE ALL (Ovid) search was conducted to ensure adequate literature availability and was validated through the retrieval of a key set of relevant studies. The search strategy has since been refined to meet the revised review scope (Appendix 1). This MEDLINE strategy will be translated to the following databases: Cochrane Central Register of Controlled Trials (Ovid), Embase and Embase Classic (Ovid), APA PsycInfo (Ovid), AMED (Ovid), AgeLine (EBSCO), CINAHL Complete (EBSCO), and Scopus. The database search strategy consists of two broad concepts: (1) NPs and (2) LTC. When functionality is available in the databases, searches will be limited to identify English language publications as restricting reviews to English language publications appears to have little impact on conclusions of reviews [27]. We will conduct the search from 1960 onwards as this marked the introduction of the NP role nationally and internationally.

A search of the grey literature will be conducted using grey literature databases (e.g., OpenGrey) and search engines (e.g., Google) to identify any relevant non-indexed literature or alternative media. The reference list of the included studies will be hand searched for additional relevant scientific and grey literature. In addition to calling upon the expertise and experience of team members, we will reach out to experts in fields related to interprofessional models of care to further identify unpublished yet relevant data.

### Stage 3: Study Selection

#### Process

Following the search, all citations will be uploaded to Covidence systemic review software and duplicates will be removed [28]. A minimum of two independent reviewers will screen all retrieved titles and abstracts against a priori inclusion and exclusion criteria to identify potentially relevant studies. Reviewers will retrieve full-text articles of potentially relevant studies/reports and two reviewers will evaluate them against the a priori criteria. Any disagreements will be resolved through consultation with other team members to reach consensus. Throughout, we will hold monthly meetings with team researchers and collaborators to discuss progress and findings. Data extraction and literature quality assessments will be managed using Covidence [28].

#### Inclusion Criteria

This scoping review will incorporate the Population-Concept-Context Framework to ensure clarity in our scope of inclusion [29]. The population represented are NPs and physicians, the concept is ‘collaborative models of care’, and the context is LTC homes.

The following literature will be included:

- The population includes NPs and physicians primary care providers. The NP role will be operationalized as graduate-prepared advanced practice nurses who assess, diagnose, and treat patients in collaboration with other healthcare professionals. The physician role will be operationalized as any clinician with a medical degree, including all specialties.
- The literature describes a collaborative model of care. We have loosely operationalized models using Davidson’s definition [30]: “An overarching design for the provision of a particular type of health care service”. Based upon our broad approach to study retrieval, the concepts of ‘collaboration’ and ‘models of care’ will be identified when reviewing the literature in this step, and manually included.
- The setting is LTC homes, operationalized as a residential, long-stay facility that provides 24/7 health and personal care.
- All methodologies of scientific literature (e.g., qualitative, quantitative, mixed, reviews, case studies, theoretical, applied, etc.).
- Published editorials and commentaries.
- Grey literature, including published and unpublished data, such as community and policy reports, government or public agency publications, conference proceedings, dissertations, practice guidelines, educational materials, etc.
- Literature published in English.
- Literature published since 1960.

#### Exclusion Criteria

The following literature will not be included

- Books and book chapters.
- The context is not described as LTC (e.g., acute care, community care, home care, transitional care programs, skilled nursing facilities, etc.).

### Stage 4: Charting the data

Data charting and literature quality assessments will be managed using Covidence to sift, categorize, and sort findings according to key issues and themes. The data charting form (Table 1) includes study characteristics, population and setting data, and the multiple components of models of care. Structure will be charted, including the characteristics of NPs and physicians in LTC homes, the LTC type, size, setting, funding models, and educational components. Process will include a description of the collaborative models of care and the specific roles of the NP and physician, strategies used for care through interprofessional collaboration, and barriers and facilitators for implementation. Outcomes will be specified for residents, staff, healthcare systems, and cost where applicable.

**Table 1:** Data charting form

**Author & Year**	**Study aim**	**Study origin & design**				
Structure						
NP Characteristics	**Physician Characteristics**	**LTC home Type** (E.g., Profit, Not-for-profit, Municipal)	**LTC home Size** (E.g., Number of beds)	**Rural or Urban setting**	**Funding of model**	**Education or Training component** (E.g., entry level training, continuing education, etc.)
Process						
Description of roles within model	**Barriers, facilitators to implementation**	**Model limitations** (E.g., resource intensive)				
Outcomes						
Resident outcomes	**Staff outcomes**	**Health systems outcomes**	**Cost outcomes**			

The charting form will be piloted on five studies by two independent analysts to ensure adequacy and consistency. Any changes to the form will be made in consultation with the team prior to extraction of data from all articles.

### Quality appraisal

The Joanna Briggs Institute’s critical appraisal tools will be used for quality appraisal of all studies included for synthesis [31]. A minimum of two appraisers will use these checklists to assess trustworthiness, relevance and results of scientific papers, opinion, and grey literature to ultimately inform data interpretation and synthesis. We will employ appraisal tools that are relevant to the study type, including qualitative, quantitative, reviews, case studies and opinion texts [31].

### Stage 5: Collating, summarizing and reporting results

To capture the extent and nature of the data retrieved, we will present it both qualitatively and quantitatively. Findings of this scoping review will be summarized with the PRISMA flowchart [26]. An overview of the categorical features of the literature will be presented numerically. We will also take a narrative synthesis approach to present the descriptive findings of the selected literature [22–25]. Using this narrative mapping exercise, a framework will be generated according to Donabedian’s structure-process-outcome model [32]. We will aim to summarize models of care, NP and physician roles, strategies for interprofessional collaboration, and associated outcomes to inform future implementation and evaluation.

### Stage 6: Consultation

Our consultation plan includes both integrated and end-of-grant knowledge translation (KT). We will engage stakeholders in a formal review of the collaborative NP/physician models and the emerging framework developed in the synthesis of the data. Stakeholders will include representatives from organizations representing NPs and physicians working in LTC; organizations representing not-for-profit, private, and municipal LTC homes, who are team members of the project. These individuals include healthcare providers, policy decision makers, and leaders of healthcare organizations. We will adopt a qualitative approach to solicit input from stakeholders to determine the relevance of the emerging framework to Ontario LTC settings and suggest ways to modify it as needed to enhance its applicability. We will share information on the collaborative NP/physician models and the framework, inviting them to discuss structures, processes, and outcomes of the emerging framework. Discussions will be audio-recorded and transcribed verbatim [33]. Transcripts will undergo content analysis by two independent members of the research team to develop themes and subthemes representative of stakeholder findings, which we anticipate will be modified iteratively through these processes.

Once data are analyzed, we will hold a meeting to present findings to the whole team and to solicit feedback on the final content before broader dissemination of findings to decision-makers within the LTC sector, and NP and physician associates. Findings from the scoping review will be presented at national and international conferences and published in peer-reviewed journals. With the support of our stakeholders, we will also disseminate fact sheets, evidence briefs, and reports targeted at specific audiences. Existing resources and platforms of our stakeholder organizations, including websites, mail-outs, and information sessions, will be used to disseminate evidence briefs to accelerate the impact of our findings.

## Discussion

This study procedure is grounded in a collaborative, interdisciplinary approach that will strengthen our understanding of NP and physician collaborative models of care within LTC homes. Findings will report on resident, staff, health systems, and cost outcomes associated with various collaborative models; synthesize findings that focus on best workforce experience for NPs and physicians; and elucidate suitability and applicability of these models from the perspective of our knowledgeable stakeholders.

Our ultimate aim is to provide decision-makers and knowledge users with high-quality, timely, and relevant evidence of the models of physician and NP collaboration in LTC homes. Consideration of the reported structures, process and outcomes of these models of care and subsequent input from stakeholders across Ontario will inform the development of a collaborative practice framework for long-term care clinical leadership. Findings of this scoping review will inform the development of a framework that when implemented, should be evaluated for its value to improvements in primary care for LTC home residents.

## Supplementary Information

**Additional file 1:** **Appendix 1.** MEDLINE search strategy

## Data Availability

The data analyzed during the current study will be available from the corresponding author on reasonable request.

## Abbreviations

KT: Knowledge Translation
LTC: Long-Term Care
MRP: Most Responsible Provider
NP: Nurse Practitioner
