# Supplementary material for: Nurse practitioner/physician collaborative models of care: a scoping review protocol
Source: BMC Geriatr. 2023 Feb 16;23:98. doi: 10.1186/s12877-023-03798-1 (PMC9934505; doi:10.1186/s12877-023-03798-1)
Supplement: Supplementary file 1 — Additional file 1: Appendix 1. MEDLINE search strategy [file 12877_2023_3798_MOESM1_ESM.docx]

## Appendix 1. MEDLINE Search Strategy

| **Concept** | **MeSH map term to subject heading** | **Keyword searches (Number of results)** |
| --- | --- | --- |
| Nurse Practitioners | Nurse Practitioners/ (MH "Nurse Practitioners") OR (MH "Gerontologic Nurse Practitioners") OR (MH "Advanced Practice Nurses") OR (MH "Advanced Nursing Practice") OR (DE "Nurse Practitioners") | Nurse Practitioner?.tw,kf,jw. (20941)  (advance? practice adj (nurs* or clinician? or professional? or provider?)).tw,kf,jw. (4580)  (NP or NPs or APN or APNS or APRN or APRNs or ARPN or ARPNs or CRNP or CRNPs or CNP or CNPs or LNP or LNPs or NPC or NPCs).tw,kf. (103811)  (nurse clinician? or nurse consultant? or nurse specialist?).tw,kf. (5348)  medical assistant?.tw,kf. (1042)  advanced clinical practitioner?.tw,kf. (70)  limited-license practitioner?.tw,kf. (3)  (non-physician adj (provider? or practitioner? or clinician? or physician?)).tw,kf. (286)  ((Mid-level or midlevel) adj (provider? or practitioner? or clinician?)).tw,kf. (629)  ("Most responsible" adj3 (provider? or practitioner? or clinician? or physician?)).tw,kf. (44)  ((Physician or MD) adj extender?).tw,kf. (406) |
| Long-Term Care | Nursing Homes/ (MH "Nursing Homes") OR (MH "Nursing Home Personnel") OR (DE "Nursing Homes" OR DE "For Profit Nursing Homes" OR DE "Nonprofit Nursing Homes" OR DE "Teaching Nursing Homes" OR DE "Nursing Home Chains" OR DE "Nursing Home Placement" OR DE "Nursing Home Standards")  Residential Facilities/ (MH "Residential Facilities")  Homes for the Aged/ (DE "Homes for the Elderly")  Long-Term Care/ (MH "Long Term Care") OR (DE "Long Term Care" OR DE "Long Term Care Administration") | "long-term care".tw,kf. (24365)  "longterm care".tw,kf. (106)  LTC.tw,kf. (4203)  ((home? or facilit*) adj2 (old age or retirement or assisted living or residential care or extended care)).tw,kw. (3529)  home? for the aged.tw,kf. (1574)  (nursing adj3 (home? or facilit* or institut* or residenc* or center* or centre*)).tw,kw. (43835)  (residenc* adj3 (elder* or old* or 'assisted living' or convalescen* or retire???? or 'long stay' or longstay or 'long term')).tw,kw. (957)  (facilit* adj3 (elder* or old* or 'assisted living' or convalescen* or retire???? or resident* or 'long stay' or longstay or 'long term')).tw,kf. (16466)  (home? adj3 (elder* or old* or 'assisted living' or convalescen* or retire???? or resident* or 'long stay' or longstay or 'long term')).tw,kf. (21495)  extended care facilit*.tw,kf. (483)  extended care home?.tw,kf. (3)  (housing adj3 (retirement or old age or senior*)).tw,kf. (439) |
